# Predictive factors for treatment success of transforaminal epidural steroid injection in lumbar disc herniation-induced sciatica

**DOI:** 10.3906/sag-1908-167

**Published:** 2020-02-13

**Authors:** Savaş ŞENCAN, Alp Een ÇELENLİOĞLU, Ruslan ASADOV, Osman Hakan GÜNDÜZ

**Affiliations:** 1 Division of Pain Medicine, Department of Physical Medicine and Rehabilitation, Faculty of Medicine, Marmara University, İstanbul Turkey; 2 Division of Pain Medicine, Department of Physical Medicine and Rehabilitation, Faculty of Medicine, Erciyes University, Kayseri Turkey; 3 Department of Radiology, Baku Medical Plaza, Baku Azerbaijan

**Keywords:** Epidural injection, herniated disc, low back pain, back pain with radiation, magnetic resonance imaging, sciatica

## Abstract

**Background/aim:**

The aim of this study was to identify predictive factors for treatment success in transforaminal epidural steroid injection in patients with lumbar disc herniation-induced sciatica.

**Materials and methods:**

A total of 219 patients who were diagnosed with unilateral sciatica and underwent transforaminal epidural steroid injections at the level of L4-5, L5-S1, or S1 neural foramina between March 2016 and May 2018 were retrospectively analyzed. The presence of transitional vertebrae and the grade of nerve root compression were evaluated by a radiologist. Data including age, sex, body mass index, duration of symptoms, injection levels, and pain scores were recorded. Pain scores were evaluated using the numerical rating scale. Treatment success was defined as a ≥50% decrease in pain scores at 3 months.

**Results:**

The study included 118 female and 101 male patients with a mean age of 43.65 ± 12.18 years. The mean duration of symptoms was 25.64 ± 2.17 weeks. Although the duration of symptoms was longer in patients for whom treatment failed, it did not reach statistical significance. Decreased pain scores at 1 h had a significant effect on treatment success (p = 0.012, odds ratio (OR): 1.015, 95% confidence interval (CI), 1.003–1.026).

**Conclusions:**

Our study results suggest that a decreased pain score at 1 h is a predictor for a favorable three-month response to transforaminal epidural steroid injection in patients with lumbar disc herniation-induced sciatica.

## 1. Introduction

Lumbar disc herniation (LDH)-induced sciatica is a common health problem with a lifelong prevalence of 12.2%–43% and an annual prevalence of 2.2%–34% [1]. Pain is triggered by mechanical compression on the dorsal root or ganglion of the herniated disc material or inflammation induced by chemokines and enzymes in the disc [2]. Several studies have shown the short-term efficacy of transforaminal epidural steroid injection (TFESI), which is commonly used in clinical practice in the treatment of sciatica [3,4]. It may exert its effect through the antiinflammatory and neural membrane stabilization effect of the steroid injection, increased blood flow of the ischemic spinal root through the local anesthetic agent, and removal of cytokines due to the washout effect from the injection material [5,6]. In addition, TFESI is a target-specific and a favorable option for delivering the injection material to the ventral epidural site where pathological alterations occur [7]. 

Despite the established short-term efficacy of TFESI for the treatment of LDH-induced sciatica, similar treatment outcomes may not be obtained in all patients. This indicates that there are several factors which affect treatment success. A number of studies have been carried out to investigate the clinical and radiological parameters which can affect TFESI outcomes [8–21]. These studies have shown that the duration of symptoms before TFESI has an effect on treatment outcomes with an inverse correlation between duration of pretreatment symptoms and treatment outcomes [11,16,21]. Some authors have demonstrated that duration of symptoms has no effect on treatment outcomes [10,18]. Spinal nerve root compression, as assessed by lumbar magnetic resonance imaging (MRI), has been associated with decreased pain scores after TFESI, and low-grade nerve root compression responds better to the treatment [9,10]. In contrast, some authors found that high-grade spinal nerve compression positively affects the treatment response or that there is no correlation between the grade of the nerve root compression and treatment outcomes [12,13]. 

To the best of our knowledge, there is a limited number of studies investigating possible factors affecting treatment outcomes in the literature. Therefore, the role of clinical and radiological parameters on TFESI outcomes has not been elucidated, and the predictive factors for treatment success are not clearly understood. In the present study we aimed to identify predictive factors for successful treatment through TFESI in patients with LDH-induced sciatica. 

## 2. Materials and methods

All patients who were diagnosed with LDH-induced sciatica, as confirmed by physical examination and lumbar MRI, and who underwent TFESI at standard doses of corticosteroids and local anesthetics between March 2016 and May 2018 were retrospectively analyzed. Of 826 patients who underwent TFESI, 219 met the inclusion criteria and were included in the study. Inclusion criteria were as follows: 18 to 65 years of age; LDH at the level of L3-4, L4-5, or L5-S1, as evidenced by MRI and TFESI, due to unilateral L4, L5, or S1 spinal nerve root compression; and complete three-month follow-up data. Exclusion criteria were as follows: prior lumbar surgery including lumbar fusion or laminectomy; lumbar spinal stenosis, spondylosis, or spondylolisthesis; local or systemic infections; inflammatory rheumatic diseases such as ankylosing spondylitis and psoriatic arthritis; history of malignancy; repeated TFESI during <3-month follow-up or missing data.

Data including age, sex, body mass index (BMI), duration of symptoms, and injection levels were recorded. Pain scores were evaluated before and at 1 h, 3 weeks, and 3 months after TFESI using the numerical rating scale (NRS). The presence of transitional vertebrae and the grade of nerve root compression were evaluated by a radiologist. Treatment success was defined as a ≥50% decrease in pain scores at 3 months [22,23]. The study protocol was approved by the ethics committee of the Marmara University, Faculty of Medicine (no.: 09.2018.591). The study was conducted in accordance with the principles of the Declaration of Helsinki. As it is routine practice for transforaminal epidural steroid injection procedures in our clinic, all patients were asked to fill out and sign the standard patient consent form prior to the procedure. The ethics committee waived the requirement for informed patient consent because patient recontact was not established for this study.

### 2.1. Radiological assessment

The radiologist who was blinded to all clinical data had 7 years of experience in spinal and musculoskeletal imaging. Cervicothoracic sagittal scout images were accepted as the gold standard for numbering the lumbar vertebral segments [24]. The vertebrae were numbered by counting caudally from C2 with cross-referencing cervicothoracic and lumbar sagittal MRI scans on the Picture Archiving and Communication System (PACS) (Carestream Health Inc., Rochester, NY, USA) workstation using the spinal-tagging properties of the software. The presence of lumbar transitional vertebrae was recorded according to vertebra count and numbering. 

The grade of nerve compression was assessed on axial T2-weighted images and sagittal T1- weighted images, respectively. For the classification of spinal nerve root compression, the modified Pfirrmann grading system was used for central and subarticular disc herniation [25]. Accordingly, grade I applies when the disc simply contacts the nerve root; grade II when the nerve root is displaced, but with preservation of periradicular cerebrospinal fluid (CSF) or fat; grade III when the periradicular CSF or fat is obliterated; and grade IV when the nerve root is morphologically distorted. Grades I and II indicate low-grade nerve root compression, while grades III and IV indicate high-grade nerve root compression. No grading for foraminal herniation was used, due to the lack of data for foraminal and extraforaminal LDH patients. 

### 2.2. TFESI procedure

All patients were placed in the prone position and supported with a pillow under the abdomen to reduce lumbar lordosis. The injection site was cleaned with povidone–iodine antiseptic 3 times and covered with a sterile dressing. The arm of the fluoroscope was rotated obliquely 10°–30° toward the region in the cranial direction, and the foramen was visualized. Local anesthesia (3 cc of 2% prilocaine) was administered to the skin and subcutaneous tissue. A Quincke 3.5-inch 22-gauge spinal needle was inserted under the intermittent guidance of fluoroscopy using the coaxial technique and advanced to the subpedicular space in the 6 o’clock direction. The needle position was confirmed through a lateral view. Following confirmation, 1–2 mL of contrast dye was given, and needle position in the epidural space was confirmed in anteroposterior and lateral views. Once adequate flow of contrast dye was achieved without vascular flow, a mixture of 80 mg of methyl prednisolone acetate, 1 cc of physiological saline, and 1 cc (0.5%) of bupivacaine was injected. There were no acute complications after the TFESI in this study.

### 2.3. Statistical analysis

Statistical analysis was performed using SPSS for Windows version 24.0 software (IBM Corp., Armonk, NY, USA). Descriptive data were expressed as mean ± standard error of measurement (SEM), number and frequency. The normality of the distribution of continuous variables was tested using the Shapiro–Wilk test. The Mann–Whitney U test was used to compare 2 independent groups with abnormally distributed data. The chi-square test was applied to analyze the relationship between 2 categorical variables. Multivariate logistic regression analysis was performed to estimate the odds ratio (OR) and 95% confidence interval (CI). Univariate and multivariate regression analyses were carried out to identify possible predictive factors for treatment success. These factors included age, sex, symptom duration, BMI, injection level, the presence of transitional vertebrae, NRS scores before injections, the grade of nerve root compression, and postprocedural 1 h NRS score decrement. A p-value of <0.05 was considered statistically significant. 

## 3. Results

Of the patients, 118 were females and 101 were males with a mean age of 43.65 ± 12.18 years. The mean duration of symptoms before injection was 25.64 ± 2.17 weeks. The most common nerve roots injected in patients were L5 (50.2%), S1 (47.5%), and L4 (2.3%). Demographic, radiological, and procedural characteristics are shown in Table 1. 

**Table 1:** Demographic, radiological, and procedural characteristics.

Variable		Value (n = 219)
Age, mean ± SEM (years)		43.65 ± 0.82
Sex	Female	118 (53.9)
	Male	111 (46.1)
BMI, mean ± SEM (kg/m²)		27.06 ± 0.29
Mean duration of symptoms (weeks)		25.64 ± 2.17
Level of injection	L4-5	5 (2.3)
	L5-S1	110 (50.2)
	S1 foramen	104 (47.5)
Nerve rootcompression (%)	Grade 1	45 (21.5)
	Grade 2	54 (25.8)
	Grade 3	50 (23.9)
	Grade 4	60 (28.7)
Transitional vertebrae	Present (%)	18 (8.2)
	Absent (%)	201 (91.8)
NRS, mean ±SEM	Before injection	7.33 ± 0.13
	Postprocedural 1 h	1.17 ± 0.13
	Week 3	3.19 ± 0.18
	Month 3	3.84 ± 0.20

BMI: body mass index; NRS: numerical rating scale. Values expressed as mean ± SEM, number, and frequency, or as otherwise indicated.

Of the 219 patients who underwent TFESI, 124 (56.6%) achieved treatment success in the 3rd month. There was no significant difference in age, sex, BMI, injection level, presence of transitional vertebrae, NRS scores before injections, and the grade of nerve root compression between patients with and without treatment success (Tables 2–3). Although the duration of symptoms was longer in patients for whom treatment failed, it remained at borderline significance (p = 0.051). There was a higher decrease in NRS pain scores at 1 h after procedure in patients for whom treatment was successful (p = 0.024). Factors with a p-value of ˂0.10 in univariate analysis were included in the multivariate binary logistic regression analysis. At the end of the analysis, symptom duration was not found to be a significant predictor for treatment success (p = 0.391). Decreased pain scores at 1 h had a significant effect on treatment success (p = 0.012, OR: 1.015, 95%, CI: 1.003–1.026) (Figure).

**Table 2:** Possible categorical variables.

		Treatment Success				
		Successful(n = 124)		Unsuccessful(n = 95)		
		n	%	n	%	p
Sex	Female	70	56.5%	48	50.5%	0.383
	Male	54	43.5%	47	49.5%	
Level of injection	S1 foramen	60	48.4%	44	46.3%	0.508
	L5-S1	60	48.4%	50	52.6%	
	L4-5	4	3.2%	1	1.1%	
Grade of nerve root compression	Low-grade	55	46.6%	44	48.4%	0.803
	High-grade	63	53.4%	47	51.6%	
Transitional vertebrae	Present	9	7.3%	9	9.5%	0.554
	Absent	115	92.7%	86	90.5%	

**Table 3:** Possible continuous variables.

	Treatment Success		
	Successful (n = 124 )	Unsuccessful (n = 95)	p
Age (years)	43.4 ± 1.14	43.97 ± 1.18	0.616
BMI	27.06 ± 0.39	27.06 ± 0.44	0.776
Duration of Symptoms (weeks)	23.65 ± 2.84	28.24 ± 3.36	0.051
NRS 1 h decrements (%)	88.4 ± 1.89	79.5 ± 2.9	0.024

BMI: body mass index; NRS: numerical rating scale.

## 4. Discussion

With the recent introduction of spinal interventional pain management modalities, the number of pain interventionalists has been on the rise bringing considerably increased treatment costs [26]. Therefore, it is of utmost importance for clinicians to identify patients in whom TFESI could be successful. Hence, predictive factors which affect the treatment outcomes positively or negatively should be established. In the present study we evaluated predictive factors for treatment success of TFESI in patients with LDH-induced sciatica. Among all parameters, only decreased pain scores at 1 h after procedure were highly correlated with treatment success at 3 months.

In another study, Inman et al. [8] evaluated the effect of epidural steroid injections on pain in patients with low back pain and reported that sex was not a predictor for decreased pain scores. This finding is consistent with our study which showed that sex had no effect on treatment outcomes. This finding has been supported by several studies in the literature [9,13,14,18]. In our study age was not a predictor for treatment success. This may be because we excluded elderly patients with degenerative pathologies such as lumbar spinal stenosis or spondylosis and only included those with LDH-induced sciatica. This finding is supported in many studies in the literature [9,11,14,18]. In contrast, Lee et al. [13] showed that mean age was higher among patients who experienced TFESI as successful, suggesting that young patients tended to have more components of the nucleus pulposus, leading to an inflammatory reaction and more resistant sciatica. However, these are short-term results. In another study, Ekedahl et al. [21] reported that a young age at treatment was a strong predictor for one-year treatment response to TFESI, while BMI was not a predictive factor for three-month and one-year treatment response. To the best of our knowledge, there is scarce literature regarding the effect of BMI on TFESI outcomes. In our study we were unable to identify BMI as a predictor for treatment outcomes during a three-month follow-up. This can be attributed to the small sample size in both studies.

In the present study we applied TFESI mainly at the L4-5 level, followed by the S1 foramen and L3-4 level. The injection level was not a predictor for treatment outcomes. This finding is consistent with our clinical observations. However, 6 patients underwent TFESI at the L3-4 level. Although further large-scale studies are needed to obtain more accurate data, our findings are consistent with the literature [9,18]. In their study, Son et al. [27] examined the effect of lumbar transitional vertebrae on the treatment response to TFESI and reported that sacralized vertebrae had an adverse effect on treatment outcomes. In the aforementioned study the authors found sacralization in 33 of 291 LDH patients. In our study we also found sacralization in 18 of 219 patients. However, the presence of transitional vertebrae did not affect treatment outcomes. This may be explained by the small sample size or the type of transitional vertebrae.

Spinal nerve root compression, as assessed by lumbar MRI, has been associated with decreased pain scores after TFESI, and low-grade nerve root compression responds better to the treatment [9,10]. Some authors have suggested that patients with high-grade spinal nerve root compression respond better to treatment [12,17]. In a retrospective study, Paidin et al. [12] reported that the higher resorption rates in patients with large disc herniation were associated with more favorable treatment responses in patients with high-grade spinal nerve root compression. In addition, Ekedahl et al. [21] found that high-grade nerve root compression was a strong predictor of adverse treatment outcomes during one-year follow-up. On the other hand, some authors have suggested that there is no correlation between spinal nerve root compression and treatment response [13,19], and this was consistent with our study findings. In our study we included patients with subarticular/central herniation and excluded those with foraminal herniation; therefore, we were unable to evaluate foraminal compression. Further multicenter, large-scale, prospective studies are required to establish a definite conclusion.

In the present study duration of symptoms was longer in patients for whom treatment was successful, indicating borderline significance. However, regression analysis revealed that it was not a predictor for treatment success. This finding is consistent with previous studies which showed no correlation between duration of symptoms and treatment outcomes [10,18]. However, several studies demonstrated that prolonged duration of symptoms adversely affected treatment response [11,21,22]. The discrepancy among studies may be caused by the different sample sizes. In our study the decreased pain scores at 1 h after procedure were highly correlated with treatment success, indicating that a greater decrease in pain scores may increase the chance of treatment success. El-Yahchouchi et al. [15] reported that immediate, post-TFESI relief of index pain at 2 weeks was weakly associated with longer term outcomes for pain relief or functional recovery. This result can be explained by their different definition of treatment success and short-term follow-up results. 

Only pain scores at 1 h after procedure, which is a postprocedural factor, were highly correlated with treatment success. Unfortunately, it is not a preprocedural factor. Therefore, it will not contribute to predicting treatment success in patients prior to performing the procedure. On the other hand, predicting treatment success at the first hour after the procedure (i.e. in a short time after the procedure) seems to be valuable for the patient and physician, as it provides the patient with objective information about the success of the procedure.

The main strength of our study is the inclusion of a homogeneous patient population with only LDH-induced sciatica and the examination of several clinical and radiological parameters. Nonetheless, the retrospective design with a short-term follow-up is the main limitation of this study. In addition, we were unable to analyze other factors, such as contrast dispersal patterns, in predicting treatment response.

In conclusion, our study results suggest that decreased pain scores at 1 h are predictors for a favorable three-month response to TFESI in patients with LDH-induced sciatica and can be a useful marker for identifying patients who would benefit from treatment. Nevertheless, further multicenter, large-scale, prospective studies are needed to elucidate possible predictive factors for treatment success.
